# Genetic Diversity of the 2009 Pandemic Influenza A(H1N1) Viruses in Finland

**DOI:** 10.1371/journal.pone.0013329

**Published:** 2010-10-20

**Authors:** Niina Ikonen, Minna Haanpää, Esa Rönkkö, Outi Lyytikäinen, Markku Kuusi, Petri Ruutu, Hannimari Kallio-Kokko, Laura Mannonen, Maija Lappalainen, Thedi Ziegler, Ilkka Julkunen

**Affiliations:** 1 Viral Infections Unit, Department of Vaccination and Immune Protection, National Institute for Health and Welfare (THL), Helsinki, Finland; 2 Department of Infectious Disease Surveillance and Control, National Institute for Health and Welfare (THL), Helsinki, Finland; 3 Department of Virology, Helsinki University Hospital, Laboratory Services (HUSLAB), Helsinki, Finland; University of Hong Kong, Hong Kong

## Abstract

**Background:**

In Finland, the first infections caused by the 2009 pandemic influenza A(H1N1) virus were identified on May 10. During the next three months almost all infections were found from patients who had recently traveled abroad. In September 2009 the pandemic virus started to spread in the general population, leading to localized outbreaks and peak epidemic activity was reached during weeks 43–48.

**Methods/Results:**

The nucleotide sequences of the hemagglutinin (HA) and neuraminidase (NA) genes from viruses collected from 138 patients were determined. The analyzed viruses represented mild and severe infections and different geographic regions and time periods. Based on HA and NA gene sequences, the Finnish pandemic viruses clustered in four groups. Finnish epidemic viruses and A/California/07/2009 vaccine virus strain varied from 2–8 and 0–5 amino acids in HA and NA molecules, respectively, giving a respective maximal evolution speed of 1.4% and 1.1%. Most amino acid changes in HA and NA molecules accumulated on the surface of the molecule and were partly located in antigenic sites. Three severe infections were detected with a mutation at HA residue 222, in two viruses with a change D222G, and in one virus D222Y. Also viruses with change D222E were identified. All Finnish pandemic viruses were sensitive to oseltamivir having the amino acid histidine at residue 275 of the neuraminidase molecule.

**Conclusions:**

The Finnish pandemic viruses were quite closely related to A/California/07/2009 vaccine virus. Neither in the HA nor in the NA were changes identified that may lead to the selection of a virus with increased epidemic potential or exceptionally high virulence. Continued laboratory-based surveillance of the 2009 pandemic influenza A(H1N1) is important in order to rapidly identify drug resistant viruses and/or virus variants with potential ability to cause severe forms of infection and an ability to circumvent vaccine-induced immunity.

## Introduction

In September 2009, the World Health Organization (WHO) recommended to include the 2009 pandemic influenza A(H1N1) virus as the H1N1 component of the trivalent, seasonal influenza vaccine for the 2010 influenza season in the southern hemisphere. In February 2010, the same recommendation was made for the 2010/2011 influenza season in the northern hemisphere. This indicates that the world-wide circulation the 2009 pandemic influenza A(H1N1) virus has not yet undergone significant antigenic and genetic changes. This stability may be attributed to the lack of pre-existing immunity in large segments of the global human population. In serosurveys, particularly elderly individuals were found to have pre-existing cross-reactive antibodies to the novel pandemic virus that were likely derived from previous infection with an antigenically related virus such as the Spanish influenza and its immediate descendant viruses that were circulating in the early decades of the 20^th^ century [1], [2]. Continued surveillance for the emergence of viruses with significant mutations is essential. Only a few months into the pandemic, viruses resistant to oseltamivir had been detected. In addition, a study from Norway indicated that an amino acid change at residue 222 of the hemagglutinin molecule may be associated with severe forms of disease [3].

**Figure 1:**
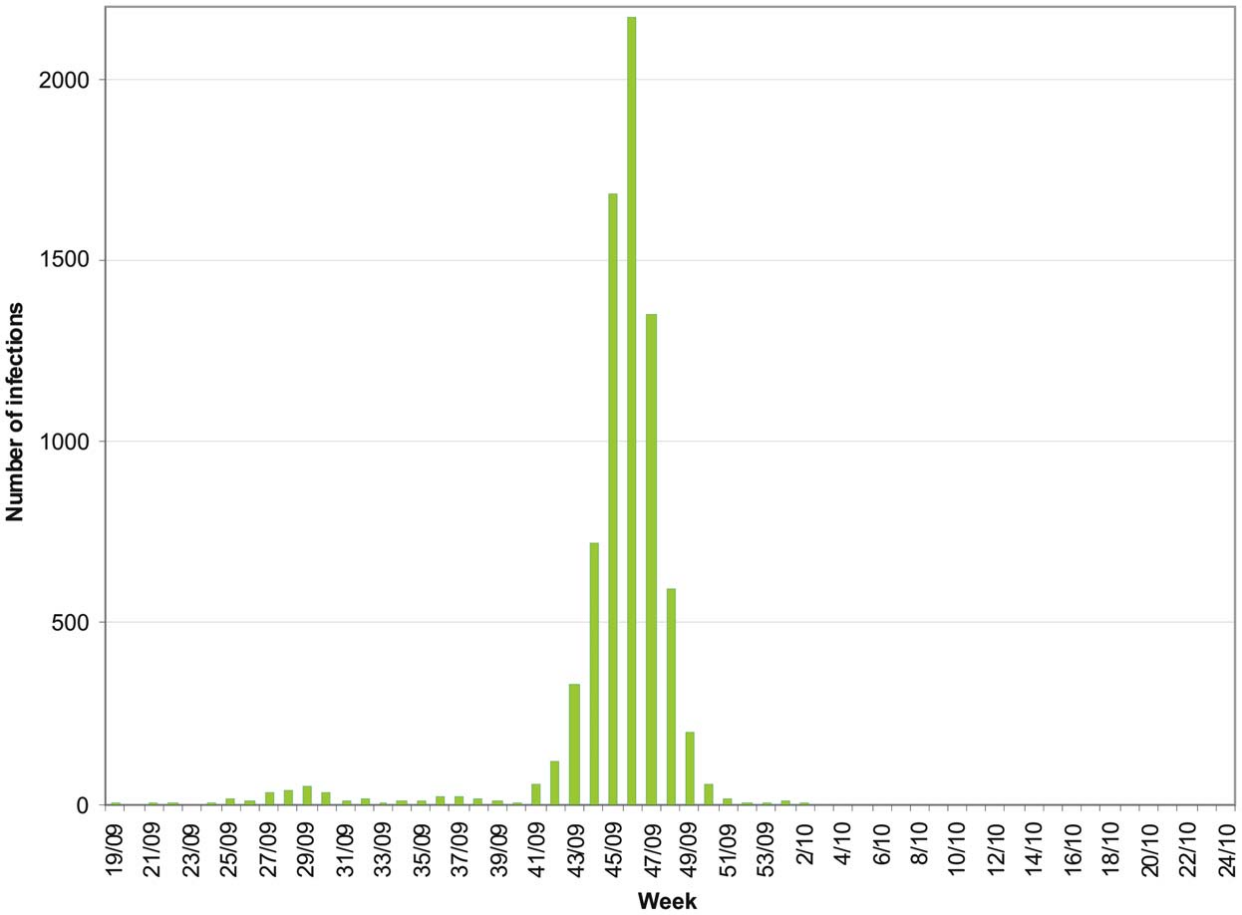
Surveillance data from May 4, 2009 to June 20, 2010 in Finland. The weekly numbers of laboratory confirmed infections of the 2009 pandemic influenza A(H1N1) viruses reported to the National Infectious Disease Registry from week 19 in 2009 to week 24 in 2010.

The novel influenza A(H1N1) virus of swine-origin emerged in humans in spring 2009. After initial reports from Mexico and the United States (USA) the virus spread rapidly to many countries. In Finland the first two infections caused by the 2009 pandemic influenza A(H1N1) virus were identified on May 10 from two individuals returning from Mexico. Between May and July 2009 nearly 90% of infections, and in August approximately 60% of infections, the 2009 pandemic influenza A(H1N1) virus was found in individuals who had recently returned from abroad. During September the first local outbreaks were recorded in garrisons and in schools in different parts of the country. In the beginning of October, the virus started to spread efficiently in the general population. Peak epidemic activity was reached late October and early November in northern, and two weeks later in southern parts of the country. Mid-December 2009 the first epidemic caused by the novel H1N1 pandemic virus was practically over in Finland (Figure 1).

Swift and open sharing of information on genetic and antigenic characteristics of the novel virus enabled rapid development of diagnostic procedures and laboratory-based surveillance throughout the world. Even minor changes in the hemagglutinin molecule may affect receptor binding specificity of the virus, and a single point mutation in the neuraminidase may render the virus resistant to oseltamivir. Continued surveillance and characterization of circulating viruses is crucial in order to identify the possible emergence of drug resistant viral strains and viruses that show significant evolution and may require the selection of future vaccine viruses. Here we present data of the genetic characteristics of viruses circulating in the Finnish population during the period from May 2009 until early February 2010, when the so far latest single infections were found.

## Results

A total of 141 pandemic influenza A(H1N1) viruses from 138 patients were analyzed in order to reveal genetic variation among viruses that were identified in Finnish patients who either had preceding travel history in countries where the 2009 pandemic influenza A(H1N1) viruses were circulating (imported infections) or who had been infected in Finland (domestic infections). Out of the 141 viruses, 48 samples were imported infections from the different continents including samples from North America (n = 23), Europe (n = 13), South America (n = 5), Asia (n = 4) and Australia and Oceania (n = 3). The remaining samples (n = 93) were chosen from domestic infections. The imported strains represented the early phases of the pandemic (May to July 2009), while the domestic infections were mainly contracted at times between August 2009 and February 2010.

### Phylogenetic and molecular analysis of the hemagglutinin (HA)

Based on the amino acid and the nucleotide sequence, the Finnish pandemic viruses were closely related to each other and to the A/California/07/2009 vaccine virus. In the HA1 region, which contains all the major antigenic epitopes of the HA molecule, the Finnish pandemic viruses differed from each other by 0 to 6 amino acids (0 to 12 nucleotides). The difference between Finnish pandemic viruses and the A/California/07/2009 virus was from 2 to 5 amino acids (3 to 10 nucleotides). The corresponding differences in the entire HA molecule were 0 to 8 amino acids (0 to 19 nucleotides) between Finnish pandemic strains, and 2 to 8 amino acids (5–14 nucleotides) as compared to the California vaccine virus.

**Figure 2:**
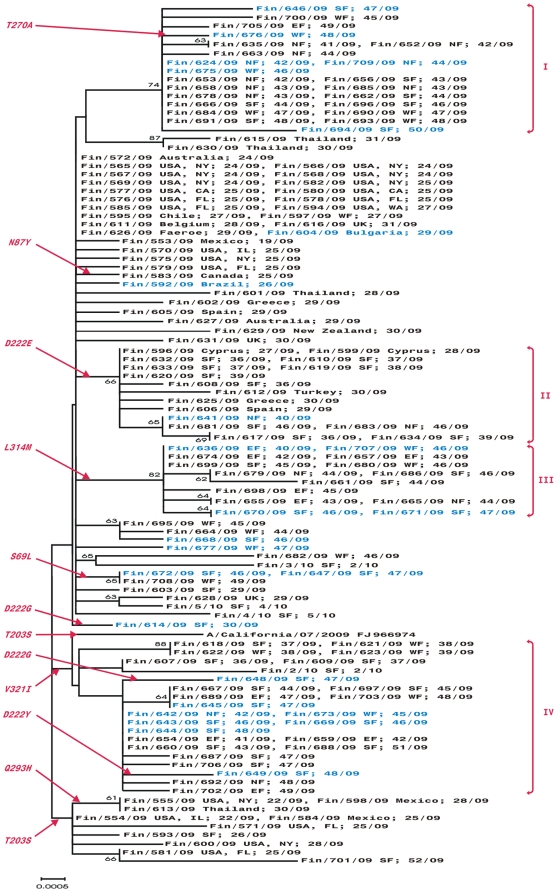
Phylogenetic tree of the HA1 domain of the 2009 pandemic influenza A(H1N1) strains from Finland. All sequences included in the phylogenetic tree were 981 nucleotides long and they covered the sequence of the HA1 portion of the HA gene without signal peptide. The horizontal lines are proportional to the number of nucleotide changes. The phylogenetic tree was constructed using the Neighbor-Joining method with Mega software version 4. In addition to the strain identification the geographic location where the infection likely occurred and the week when the sample was collected are shown. The country, and the state (in cases where the disease was contracted in USA) where the infection has been contracted is indicated. In addition, the following abbreviations are used: SF – Southern Finland, EF – Eastern Finland, WF – Western Finland, NF – Northern Finland (including Oulu and Lapland districts). Viruses identified in patients suffering from a severe infection (including fatal infections) are marked in blue.

The phylogenetic tree of HA1 nucleotide sequences of the Finnish pandemic strains and the vaccine strain is shown in Figure 2. The majority of the early, obviously imported viruses appear at the base of the tree. The domestic strains evolved further from the early imported strains and clustered into four groups numbered as I to IV (Figure 2). No clear distribution of viruses according to geographical or temporal occurrence could be observed. Group II included, together with domestic strains, viruses from the early phases of the pandemic, imported by individuals with recent travel to Cyprus, Greece, Turkey and Spain. Also viruses identified in patients suffering from severe or fatal infections were scattered throughout the phylogenetic tree. One of the characteristic differencies between the epidemic viruses and the A/California/07/2009 vaccine virus occurred at residue 203, where only 10 of the analyzed viruses had this serine (S) residue conserved. All other viruses had a change to threonine (T) at this residue. In the HA1 region at residue 69, a change from serine (S) to leucine (L) was identified in 3 viruses. The amino acid change from aspartic acid (D) to glycine (G) at residue 222 was found in two viruses and a corresponding change from aspartic acid (D) to tyrosine (Y) at the same residue was found in one additional virus. All these three changes, however, occurred as a mixture of the new amino acid and conserved aspartic acid (D). In 16 viruses a change from aspartic acid (D) to glutamic acid (E) was observed at residue 222, and these viruses form the group II in the phylogenetic tree (Figure 2). A change from glutamine (Q) to histidine (H) at residue 293 was found in three viruses identified in patients who had returned from New York, Mexico, and Thailand, respectively. Group III consisted of 14 viruses that had an amino acid change from leucine (L) to methionine (M) at residue 314. The majority of analyzed viruses (110/137) had an amino acid change from isoleucine (I) to valine (V) at residue 321. Viruses that have retained the isoleucine (I) at residue 321 form group IV. In addition, an amino acid change from glutaminic acid (E) to lysine (K) at residue 374 in the HA2 region was detected in three viruses. Table 1 lists amino acid changes that may be associated with severe disease outcome observed by us and others [3], [4], [5], [6]. The phylogenetic tree based on the entire HA gene region is presented in supporting materials (Figure S1).

**Table 1:** Relation of amino acid changes in the hemagglutinin to clinical outcome.

	Clinical outcome^a^			
Genotype of HA position	Mild	Severe or fatal	All cases	P-value^c^
222 D	86.5% (96/111)	82.6% (19/23)	85.8% (115/134)	p = 0.742
222 G	0% (0/111)	8.7% (2/23)	1.5% (2/134)	p = 0.028
222 Y	0% (0/111)	4.3% (1/23)	0.7% (1/134)	p = 0.172
222 E	13.5% (15/111)	4.3% (1/23)	11.9% (16/134)	p = 0.305
293 Q	97.3% (108/111)	100% (23/23)	97.8% (131/134)	
293 H	2.7% (3/111)	0% (0/23)	2.2% (3/134)	p = 1
321 I	17.1% (19/111)	34.8% (8/23)	20.1% (27/134)	
321 V	82.9% (92/111)	65.2% (15/23)	79.9% (107/134)	p = 0.083
374 E	98.1% (102/104)	95.5% (21/22)	97.6% (123/126^b^)	
374 K	1.9% (2/104)	4.5% (1/22)	2.4% (3/126^b^)	p = 0.441

a Percentage and number of patients of each genotype are presented in both clinical categories.

b The entire hemagglutinin sequence (HA2 portion) was not available from all patients.

c The statistical significance between the number of mild and severe cases was calculated by the Fisher's exact test.

**Figure 3:**
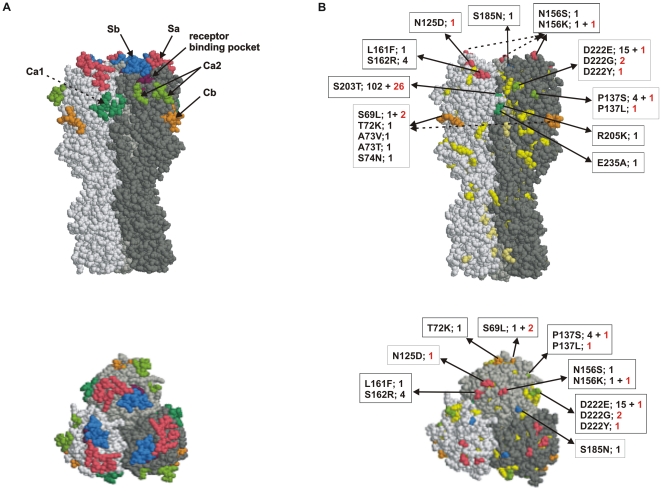
Amino acid differences in the HA between the Finnish pandemic viruses and the vaccine strain A/California/07/2009. **A.** The trimeric HA molecule with previously identified H1 protein-related antigenic sites (Sa in pink, Sb in blue, Ca1 in darker green, Ca2 in lighter green and Cb in orange) of influenza A(H1N1) viruses and with the receptor binding pocket (purple) is presented in side and from top view. Different monomers are shown in various shades of grey color. The structure is based on the 3-dimensional structure of A/South Carolina/1/18 (RCSB Protein bank accession number 1ruz) HA molecule, which is genetically the closest resolved H1 structure as compared to the 2009 pandemic H1 molecule. **B.** The amino acid differences between the HA molecules of the Finnish pandemic viruses and the A/California/07/2009 vaccine virus are shown in the trimeric HA structure. Amino acid changes in antigenic sites are colored as in panel A. Other changes (apart from antigenic sites) in the HA1 region are shown in yellow and in the HA2 region in gold. Changes in antigenic sites are illustrated by the amino acid residue number, the amino acids that have changed, and in addition, the number of viruses found to contain the respective amino acid (numbers in red denote severe infections).

Figure 3 illustrates the amino acid changes between the Finnish pandemic viruses and the vaccine virus in a three-dimensional model of the HA molecule. Almost all amino acid changes on the HA molecule were located on the surface of the molecule. Some of the changes accumulated in antigenic sites, Ca1, Ca2, Cb, Sa and Sb [7] and these viruses were derived from patients suffering both mild or severe infections.

### Phylogenetic and molecular analysis of the neuraminidase (NA)

**Figure 4:**
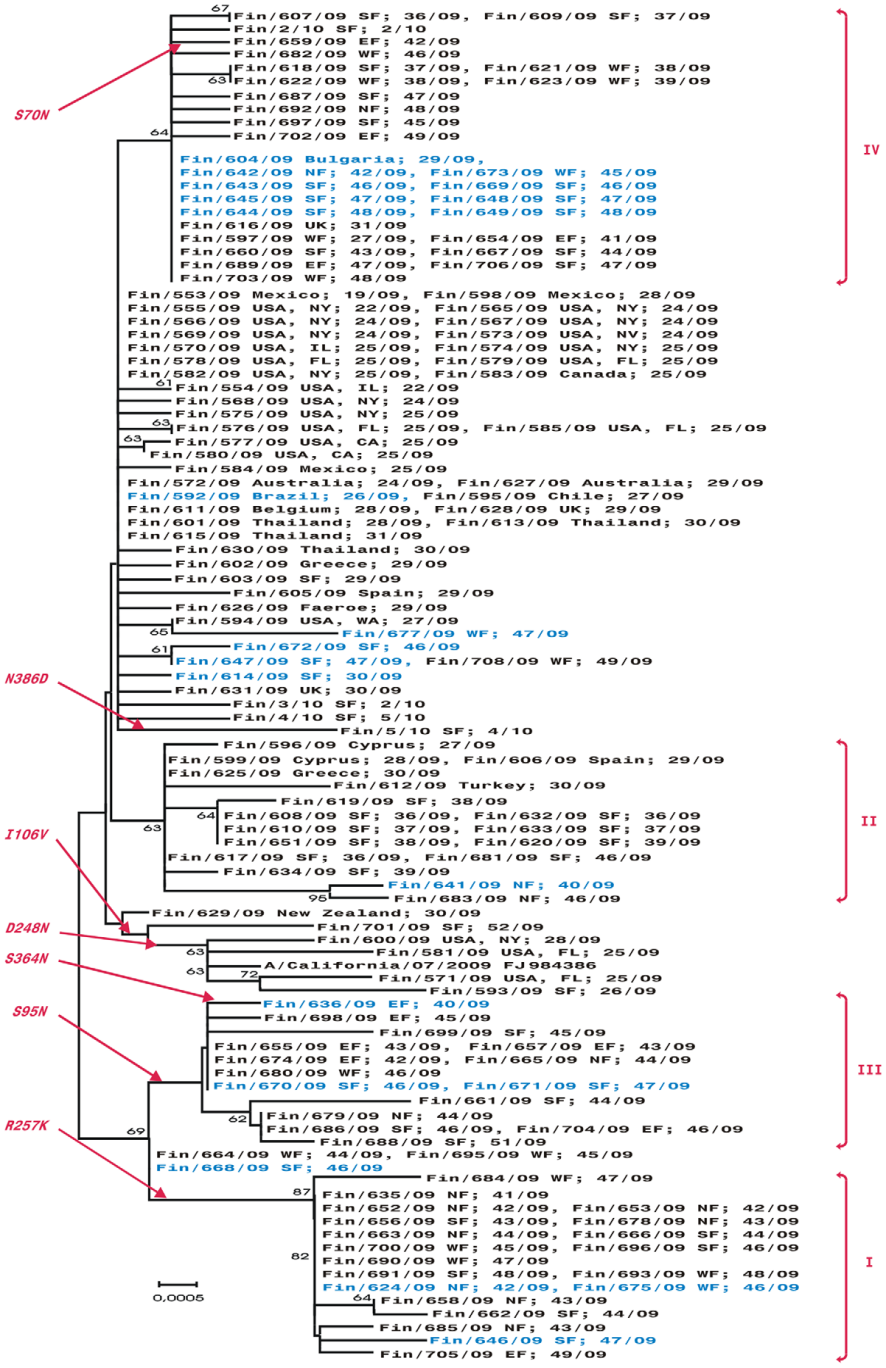
Phylogenetic tree of the NA of the 2009 pandemic influenza A(H1N1) strains from Finland. All sequences of genes included in the phylogenetic tree were 1401 nucleotides long. The horizontal lines are proportional to the number of nucleotide changes. The phylogenetic tree was constructed using the Neighbor-Joining method with Mega software version 4. In addition to the strain identification the geographic location where the infection likely occurred and the week when the sample was collected are indicated. The country, and the state (in cases where the disease was contracted in USA) where the infection has been contracted is indicated. In addition, the following abbreviations are used: SF – Southern Finland, EF – Eastern Finland, WF – Western Finland, NF – Northern Finland (including Oulu and Lapland districts). Viruses identified in patients suffering from a severe infection (including fatal infections) are marked in blue.

The NA of Finnish pandemic viruses differed from each other by 0 to 7 amino acids (0–15 nucleotides), and from the A/California/07/2009 virus by 0 to 5 amino acids (3–12 nucleotides). In the phylogenetic tree of the NA gene (Figure 4), the Finnish viruses cluster into the same four groups (Groups I–IV) as in the HA tree. The only two exceptions were A/Finland/604/2009 (imported from Bulgaria) and A/Finland/616/2009 (imported from the United Kingdom). Both viruses were located in Group IV of the NA phylogenetic tree, while they were at the basis of the HA tree together with other viruses identified from early imported infections. Viruses obtained from severe infections were scattered throughout the phylogenetic tree of the NA gene. None of the analyzed viruses had the amino acid change from histidine (H) to tyrosine (Y) at residue 275, which would render the virus resistant to oseltamivir. All 15 viruses of Group III had an amino acid change from serine (S) to asparagine (N) at residue 95. Five viruses clustered closely with the A/California/07/2009 virus, which had a valine (V) at residue 106. This valine was changed to isoleucine (I) in all other viruses. In addition, four of these five viruses had asparagine (N) at residue 248 as in the vaccine virus, while in all other viruses this amino acid was changed to aspartic acid (D). Group I was characterized by a change from arginine (R) to lysine (K) at residue 257.

**Figure 5:**
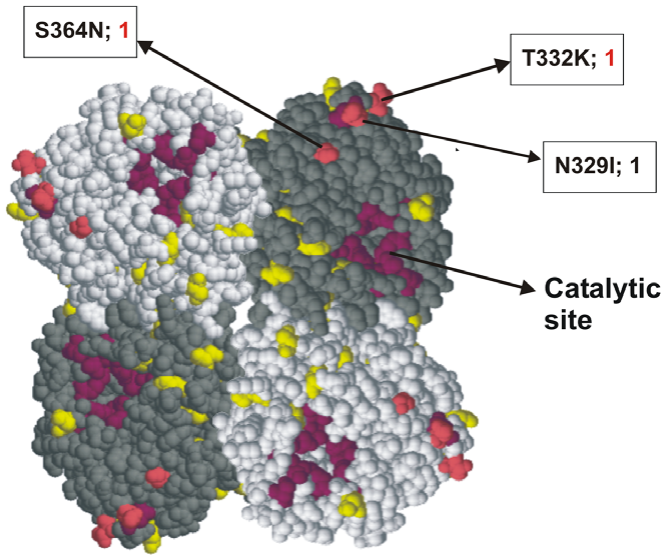
Amino acid differences in the NA between the Finnish pandemic viruses and the A/California/07/2009. The amino acid differences are located in the tetrameric NA structure. Amino acid changes in antigenic sites are colored in pink and changes in other regions in yellow. The catalytic site is marked in purple. Changes in antigenic sites are illustrated by the amino acid residue number, the amino acids that have changed, and in addition, the number of viruses found with this change is indicated (numbers in red denote severe cases). The structure is based on the 3-dimensional NA structure of A/Brevig Mission/1/1918 (RCSB Protein Bank accession number 3beq) virus.

In Figure 5, amino acid changes between the Finnish viruses and the vaccine viruses are shown in a three-dimensional model of the NA molecule. As in the HA, most amino acid changes were located on the surface of the NA molecule and these changes cover a considerable surface area of the molecule. Only three changes were found in antigenic sites [8].

### Glycosylation sites of the HA and NA molecules

Since glycosylation can potentially affect the antigenic properties of influenza A virus we analyzed the changes in the potential N-linked glycosylation sites of the pandemic virus strains. Eight potential N-glycosylation sites are found in the HA molecule of the A/California/07/2009 virus, six of which reside in the HA1 and the remaining two in the HA2 region. All but one of the Finnish pandemic viruses had retained all the potential 8 N-glycosylation sites. The one exception (A/Finland/583/2009) had lost a glycosylation site with a change at amino acid residue 87 from asparagine (N) to tyrosine (Y).

The A/California/07/2009 vaccine virus has 8 potential glycosylation sites in its NA molecule. Altogether three viruses showed changes in their potential N-glycosylation sites of the NA molecule. A/Finland/659/2009 virus had lost a glycosylation site (residues 68–70) by replacing a serine (S) at residue 70 by asparagine (N) which, in turn, lead to the formation of a novel potential N-glycosylation at residues 70 to 72 by changing the sequence from SNT to NNT. Another virus (A/Finland/5/2010) has lost a glycosylation site at residues 386–388, and in one additional virus (A/Finland/636/2009) a ninth potential N-glycosylation site was formed at position 364–366.

### Evolutionary rates of the HA and NA

**Figure 6:**
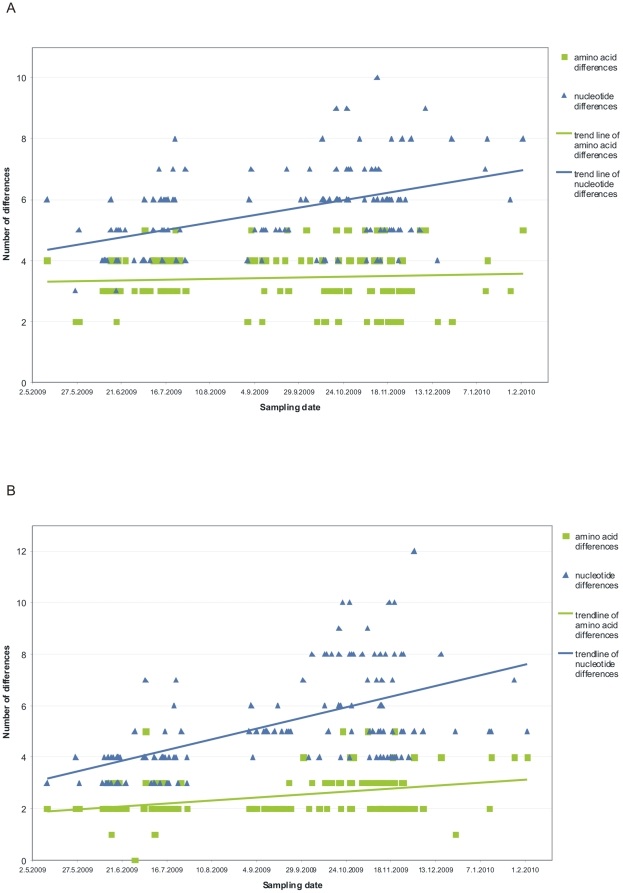
Evolutionary rates of the HA1 and NA genes. The number of nucleotide and amino acid changes between the HA1 (panel A) and NA (panel B) of Finnish pandemic viruses and the vaccine strain A/California/07/2009 are shown as a function of time. The trend lines in both pictures have been drawn using Microsoft Office Excel 2003. The numbers of nucleotide changes are shown in blue and in amino acid changes in green.

Next we calculated the evolutionary rates of the HA and NA genes. Figure 6 shows the number of nucleotide and amino acid changes in the HA1 (Figure 6A) and NA (Figure 6B) as a function of time. Viruses identified between May 2009 and February 2010 showed several nucleotide changes in their HA1 and the NA genes as compared to the A/California/07/2009 vaccine virus. The first viruses identified in Finland already showed 3 to 6 nucleotide changes in the HA1 gene, and 3 to 4 nucleotide changes in the NA gene as compared to the A/California/07/2009 virus. Thereafter in Finnish strains nucleotide changes occurred linearly as a function of time. The HA1 region accumulated 3–10 and the NA gene 3–12 nucleotide changes, respectively. Since most of the nucleotide changes in the HA1 and NA genes were silent, the rate of amino acid changes was clearly lower than the changes at nucleotide level. Data of the entire HA gene is presented in supporting materials (Figure S2).

### Characteristics of viruses from three severe clinical cases

Two subsequent clinical specimens were available from each of three patients suffering from a severe form of infection due to the pandemic virus. For two of these patients the outcome was fatal, while one suffered from a severe pneumonia that required intensive care and respirator treatment and lead to the recovery of the patient. The two samples (A/Finland/624/2009 and A/Finland/709/2009) from the first patient were taken 11 days apart. In the HA1 region, the two viruses were identical, but they differed by amino acid change from phenylalanine (F) to phenylalanine/serine (F/S) mix at residue 397 in the HA2 region. The two viruses from the second case (A/Finland/670/2009 and A/Finland/671/2009) were taken 5 days apart from each other and had identical HA1 and NA sequences. The specimens from the third case (A/Finland/675/2009 and A/Finland/676/2009) were taken 11 days apart and the viruses differed by two amino acids in the HA, one of them in the signal peptide (N16T) region and the other one in the HA1 region at residue 270 with a change from threonine (T) to alanine (A).

## Discussion

Based on nucleotide sequence data, viruses circulating in Finland during the first 9 months after the emergence of the novel 2009 pandemic influenza A(H1N1) virus differed relatively little from each other and from the A/California/07/2009 vaccine strain. In the globular head region of the HA, the most varying part of the molecule, homology at the amino acid level between the Finnish pandemic strains ranged from 98.3% to 100%. In the same region of the HA molecule, similarity between the Finnish pandemic viruses and the California vaccine strain were between 98.5% and 99.4%. Corresponding homologies for the entire HA region were 98.6%–100% among Finnish viruses, and 98.6%–99.6% between Finnish pandemic viruses and the vaccine strain. Also NA molecules were highly conserved. The Finnish pandemic viruses were 98.5%–100% identical, and homologies between the Finnish pandemic viruses and the California vaccine strain were 98.9% to 100%.

Some of the observed amino acid changes were located in the critical antigenic sites (Figures 3 and 5) of the HA and NA molecules. For some of the amino acid changes detected by us and others [3], [4], [5], [6] an association with severe illness may be assumed (Table 1). The amino acid change from aspartic acid (D) to glycine (G) at residue 222 of the HA1 was first reported for pandemic H1N1 viruses from Norway [3]. This amino acid is located in the antigenic site Ca2 in the immediate vicinity of the receptor binding pocket and it may influence binding preferences of the virus [9], [10]. It has been speculated that the binding preference of the 2009 pandemic virus may change from the cells of the upper respiratory tract to those found in lower parts of the lungs [10]. In previous reports [3], [11], [12] as well as in our samples, this amino acid change presented as a mixture between wild type virus and mutated viruses. This amino acid change has previously been associated with severe infection [3]. Similarly, in our study the two viruses with D222G change and the virus with the D222Y change were linked to severe or even fatal illness. Viruses with HA molecule D222G, D222N, and D222E changes have been found in many countries [13], but to our knowledge the D222Y amino acid change has not yet been reported by others. Thus, the two viruses with the D222G change represented 8.7% of the 23 severe infections in the present study. As in the study by Kilander et al. [3] this frequency reaches statistically significant level (p = 0.028).

Glinsky as well as Melidou and coworkers have found that viruses with an amino acid change from glutamine (Q) to histidine (H) at residue 293 may increase disease severity [4], [5]. This mutation was seen in viruses obtained from three of our patients. However, all three patients suffered from a mild form of infection, thus our analysis does not support the concept that Q293H mutation is readily associated with a more severe form of infection. The majority of viruses in our study (80.3%) had an amino acid change from isoleucine (I) to valine (V) at residue 321 in the HA1. This mutation has also frequently been observed in viruses isolated in northern Greece where this change has been found in 93% of the viruses analyzed [5]. In their study, some of the viruses that retained the isoleucine were associated with severe influenza infection. Accordingly, in our study eight of the 27 viruses that exhibited isoleucine (I) at residue 321 were identified in patients who had a severe illness. These viruses represent 34.8% of patients who had severe or even fatal outcome of the illness, which, however, does not quite reach statistical significance (p = 0.083). The amino acid change from serine (S) to threonine (T) at residue 203 in the antigenic site Ca1 of the HA1 was found in over 90% of the Finnish pandemic viruses, slightly more often as compared to the Greek viruses [5]. Viruses identified in Singapore and elsewhere showed an amino acid change from glutamic acid (E) to lysine (K) at residue 374 in the HA2 region and this change was associated with a more severe clinical picture in some patients. In the same study it was speculated that this change could influence the fusion properties of the HA molecule and thus may affect the pathogenicity of the virus [6]. We found this mutation in 3 out of 127 viruses for which the entire HA sequence was obtained, one of which was identified in a patient suffering from ARDS. In our study, significant associations to severe illness could only be demonstrated for the amino acid change to glycine at residue 222, which is in agreement with the findings of Kilander et al [3].

During the first year of the pandemic, neuraminidase inhibitors were liberally used especially in pregnant women or in patients who suffered from a severe form of infection or who had an underlying medical condition. Also in Finland oseltamivir was actively used to treat influenza patients. None of the viruses described in the present study had the amino acid change from histidine (H) to tyrosine (Y) at residue 275 of the NA molecule, a change that is commonly associated with resistance to oseltamivir.

The assumed three-dimensional structures of the HA and NA molecules enabled us to map the amino acid changes found in our viruses to the putative antigenic sites of the viral surface glycoproteins. The HA structure of the Spanish influenza virus A/South Carolina/1/18 and the NA structure of A/Brevig Mission/1/1918 were used, since the corresponding HA and NA genes are genetically the closest ones to the present pandemic viruses [1]. The antigenic sites of H1 type HA molecule have been well identified [7] (Figure 3A) and based on this data some of mutations found in circulating Finnish pandemic influenza A virus strains accumulated in the major antigenic sites. At present we do not know which of the amino acid changes located in different antigenic sites influence the formation of neutralizing or hemagglutinin inhibiting antibodies. This is evidently a topic of future analysis so that the immunological consequence of each mutation on the surface of the HA molecule could be analyzed. Similar structural analysis of the NA molecule revealed that some of the amino acid changes we observed in the NA molecule were located near the catalytic site of the neuraminidase (Figure 5). Of the mutations found on the surface of the NA molecule, at least 3 amino acid changes were located in antigenic sites [8] (Figure 5). At present, the antigenicity of NA is less well characterized as compared to HA molecule and the contribution of neuraminidase-specific antibodies in virus neutralization is still a topic of further studies.

The relatively high number of virus strains characterized in our study enabled us to estimate the evolutionary rates of HA and NA genes. During the 9 month observation period, when pandemic viruses were identified in Finland, the evolution rate at the amino acid level was estimated to be approximately 1.5% for the HA1 molecule and 1.1% for the NA molecule, respectively (Figure 6). This observed evolutionary speed is somewhat higher than what has previously been calculated for seasonal H3N2 viruses [14]. However, during the first year of circulation, mutations in the surface glycoproteins of the pandemic virus may accumulate at random until a most fit variant has established itself and further evolution takes a more linear rate. None of the mutations thus far detected in Finnish pandemic viruses gave rise to a dominant strain with high epidemic potential or replication advantage, and the evolutionary rate in the viruses was relatively low. The future years will show whether the evolutionary speed of the 2009 pandemic virus will increase from that seen during the first pandemic year. An additional interesting finding was that a novel glycosylation site was introduced in the NA molecule of one of our viruses. Similar changes could also occur in the HA which could, in turn, lead to alterations in the antigenicity and immunogenicity of the virus.

Vaccines and antiviral drugs play an important role in the prevention and treatment of influenza virus infections. In order to select suitable vaccine strains and to detect possible emergence of drug-resistant viruses the characterization of circulating viruses must be continuously performed. Considering the number of amino acid changes and their sites within the structures of the HA and NA molecules of the 2009 pandemic influenza A(H1N1) virus, it is likely that vaccination with the A/California/07/2009 or infection with the pandemic virus will provide protective immunity at least for some time to come. However, forthcoming antigenic analyses of the pandemic viruses and clinical vaccination studies by us and others will provide more information on the evolution of the pandemic virus and the protective efficacy of the present pandemic virus vaccine.

## Materials and Methods

### Clinical samples and epidemic data

The first infections of the 2009 pandemic influenza A(H1N1) were identified in Finland on May 10, 2009. During the period from May 4, 2009 and June 20, 2010, (weeks 19/09–24/10) a total of 7,669 laboratory confirmed infections of the 2009 pandemic influenza A(H1N1) viruses were reported to the National Infectious Disease Registry coordinated by the National Institute for Health and Welfare (THL). Clinical samples from suspected cases as well as some laboratory confirmed infections were sent to the National Influenza Centre at THL Helsinki for confirmation and detailed analysis. The clinical samples have been collected for routine virological laboratory diagnosis, requested by physician examining and treating the patients. Based on national laws ethical permissions are not required for specific microbiological diagnostics, treatment of the patients and further characterization of the viruses. All samples were coded and tested anonymously and further antigenic and genetic characterization of circulating influenza viruses was carried out at Viral Infections Unit at THL, which also functions as a National Influenza Centre. Patient information is stored according to national regulations and access to such data is restricted.

The detection of the 2009 pandemic influenza A(H1N1) virus was carried out by standard real-time RT-PCR procedures with specific primers for HA and NS genes. Primer sequences for real-time RT-PCR are available on request. For the present study 141 positive virus samples collected from 138 patients were chosen. They represented both mild (n = 115) and severe (n = 23) infections (pneumonia with or without acute respiratory distress syndrome (ARDS), and patients requiring intensive care that lead to recovery or to fatal outcome). Also geographic origin and temporal distribution of samples were considered in the selection of the samples that were included in the analysis. From 48 of the 138 patients previous travel history was also known. To study the genetic variation of the 2009 pandemic influenza A(H1N1) viruses since its emergence and during the first wave in Finland, the nucleotide sequences of the complete HA gene (1701 nucleotides, 567 amino acids) from 127 viruses and the HA1 region (1032 nucleotides, 344 amino acids) from additional 10 viruses were generated. The NA gene sequences (1401 nucleotides, 467 amino acids) from 137 viruses were also obtained. The sample collection date of the strains selected to the phylogenetic and molecular analysis covered the period from May 10, 2009 to February 2, 2010. The GenBank accession numbers of the HA sequences are GQ183633, GQ283488, GQ283493, GU292341, HQ228025–HQ228147 and HQ234707–HQ234716 and those of NA sequences are GQ183634, GQ283487, GQ283492 and HQ247604–HQ247737. The GenBank hemagglutinin and neuraminidase gene accession numbers for A/California/07/2009 vaccine virus were FJ966974 and FJ984386, respectively.

### Gene sequence analysis and phylogenetic analysis

RNA was isolated from a 100 µl aliquot of combined nasal and throat specimens using RNeasy Mini Kit (Qiagen GmbH, Hilden, Germany) with QIAcube automated purification instrument according to the manufacturer's instructions. In three occasions, total cellular RNA was isolated from viruses cultured in MDCK cells or embryonated hen's eggs. The viral RNA was reverse transcribed into cDNA with SuperScript™ III reverse transcriptase (Invitrogen, Carlsbad, USA) according to the manufacturer's instructions. The resulting DNA was amplified using nested PCR. The amplified products were purified with QIAquick PCR Purification Kit (Qiagen GmbH, Hilden, Germany). Sequencing of both DNA strands was carried out using an ABI Prism Big Dye Terminator Cycle Sequencing Ready Reaction Kit with an ABI 3730 automated DNA sequencer (Perkin Elmer Applied BioSystems, Foster City, CA). Primer sequences for HA and NA amplification and for sequencing are available on request. Sequence data was analyzed and fragments were assembled using Sequencher version 4.7 (Gene Codes Corporation, Ann Arbor, USA). Mega software version 4 [15] (Molecular Evolutionary Genetics Analysis) was used in sequence comparisons of HA and NA and for the construction of the phylogenetic trees. The phylogenetic trees were generated using the Neighbor-joining method [16] with the maximum composite likelihood model [17]. Bootstrapping was performed with 1000 duplicates [18]. All HA residues are numbered omitting the signal peptide sequence. In the numbering of NA residues, the signal peptide sequence was included.

### Structural analysis of HA and NA molecules

The three-dimensional structures of the HA and NA were used to locate the amino acid differences between the Finnish 2009 pandemic viruses and the A/California/07/2009 vaccine strain. Because the HA and NA of the 2009 pandemic influenza A(H1N1) virus resembles more those of the Spanish influenza virus [1] than of recent seasonal human H1N1 viruses, the three-dimensional structures of HA of A/South Carolina/1/18 (RCSB Protein Bank accession number 1ruz) and NA of A/Brevig Mission/1/1918 (RCSB Protein Bank accession number 3beq) were selected for the structural comparisons. The molecular models presented here were constructed using RasMol Molecular Graphics software version 2.7.3 [19].

### Statistical analysis of disease association with certain amino acid changes on the hemagglutinin molecule

Certain amino acid changes in the hemagglutinin molecule observed in the present study and by others [3], [4], [5], [6] have been found in severe cases (Table 1). Statistical power of such an association has been calculated by Fisher's exact test (http://www.graphpad.com/quickcalcs/contingency1.cfm).

## Supporting Information

Figure S1 Phylogenetic tree of entire HA gene of the 2009 pandemic influenza A(H1N1) strains from Finland. All sequences included in the phylogenetic tree were 1701 nucleotides long. The horizontal lines are proportional to the number of nucleotide changes. The phylogenetic tree was constructed using the Neighbor-Joining method with Mega software version 4. In addition to the strain identification the geographic location where the infection likely occurred and the week when the sample was collected are shown. The country, and the state (in cases where the disease was contracted in USA) where the infection has been contracted is indicated. In addition, the following abbreviations are used: SF - Southern Finland, EF - Eastern Finland, WF - Western Finland, NF - Northern Finland (including Oulu and Lapland districts). Viruses identified in patients suffering from a severe infection (including fatal infections) are marked in blue.
(0.92 MB TIF)

Figure S2 Evolutionary rate of entire HA gene. The number of nucleotide and amino acid changes between the HA of Finnish pandemic viruses and the vaccine strain A/California/07/2009 are shown as a function of time. The trend lines have been drawn using Microsoft Office Excel 2003. The number of nucleotide changes are shown in blue and in amino acid changes in green.
(0.48 MB TIF)
