# Supplementary figures and images for: Genetic Diversity of the 2009 Pandemic Influenza A(H1N1) Viruses in Finland
Source: PLoS One. 2010 Oct 20;5(10):e13329. doi: 10.1371/journal.pone.0013329 (PMC2958116; doi:10.1371/journal.pone.0013329)

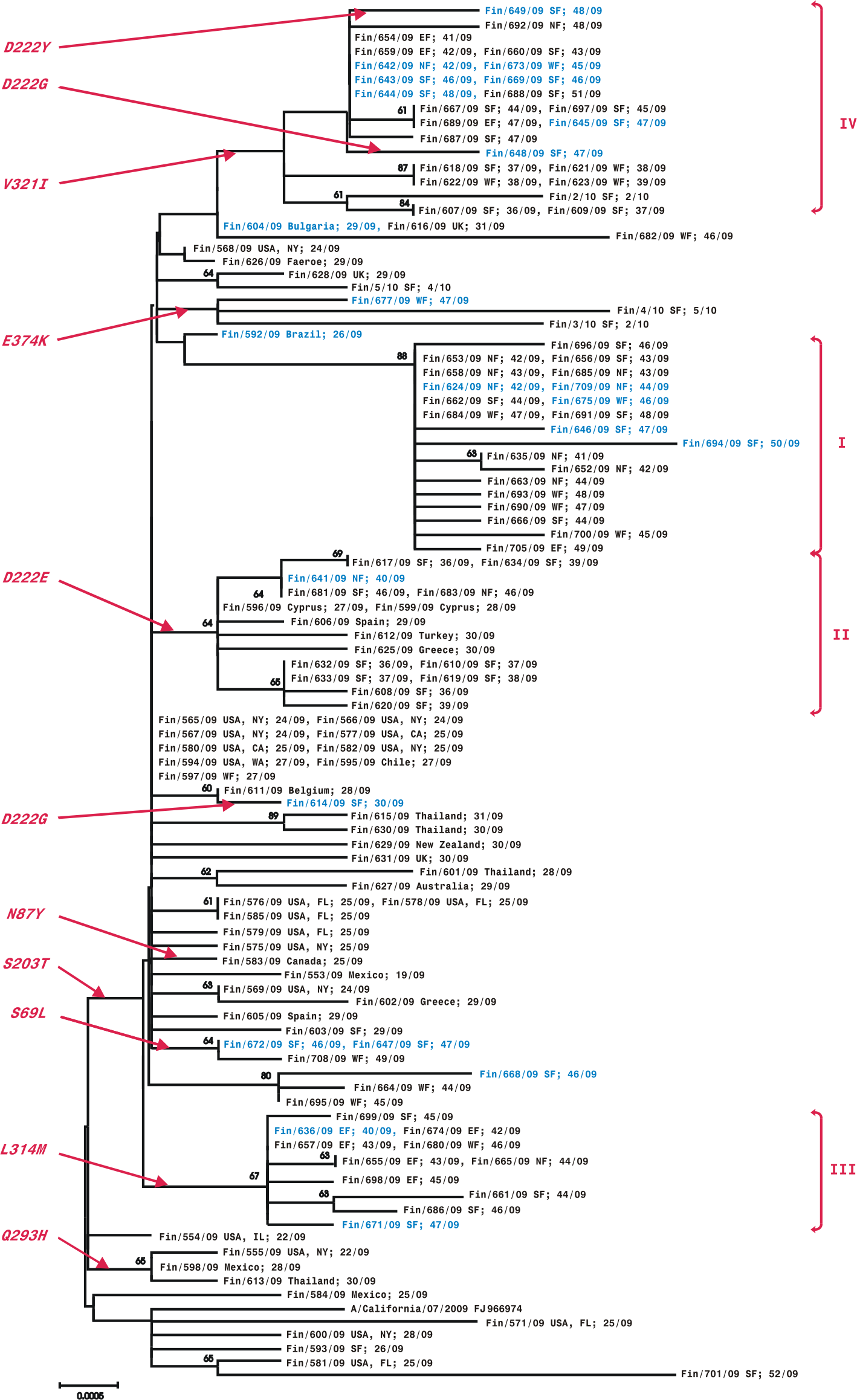

Supplement: Figure S1 — Phylogenetic tree of entire HA gene of the 2009 pandemic influenza A(H1N1) strains from Finland. All sequences included in the phylogenetic tree were 1701 nucleotides long. The horizontal lines are proportional to the number of nucleotide changes. The phylogenetic tree was constructed using the Neighbor-Joining method with Mega software version 4. In addition to the strain identification the geographic location where the infection likely occurred and the week when the sample was collected are shown. The country, and the state (in cases where the disease was contracted in USA) where the infection has been contracted is indicated. In addition, the following abbreviations are used: SF - Southern Finland, EF - Eastern Finland, WF - Western Finland, NF - Northern Finland (including Oulu and Lapland districts). Viruses identified in patients suffering from a severe infection (including fatal infections) are marked in blue. (0.92 MB TIF) [file pone.0013329.s001.tif]

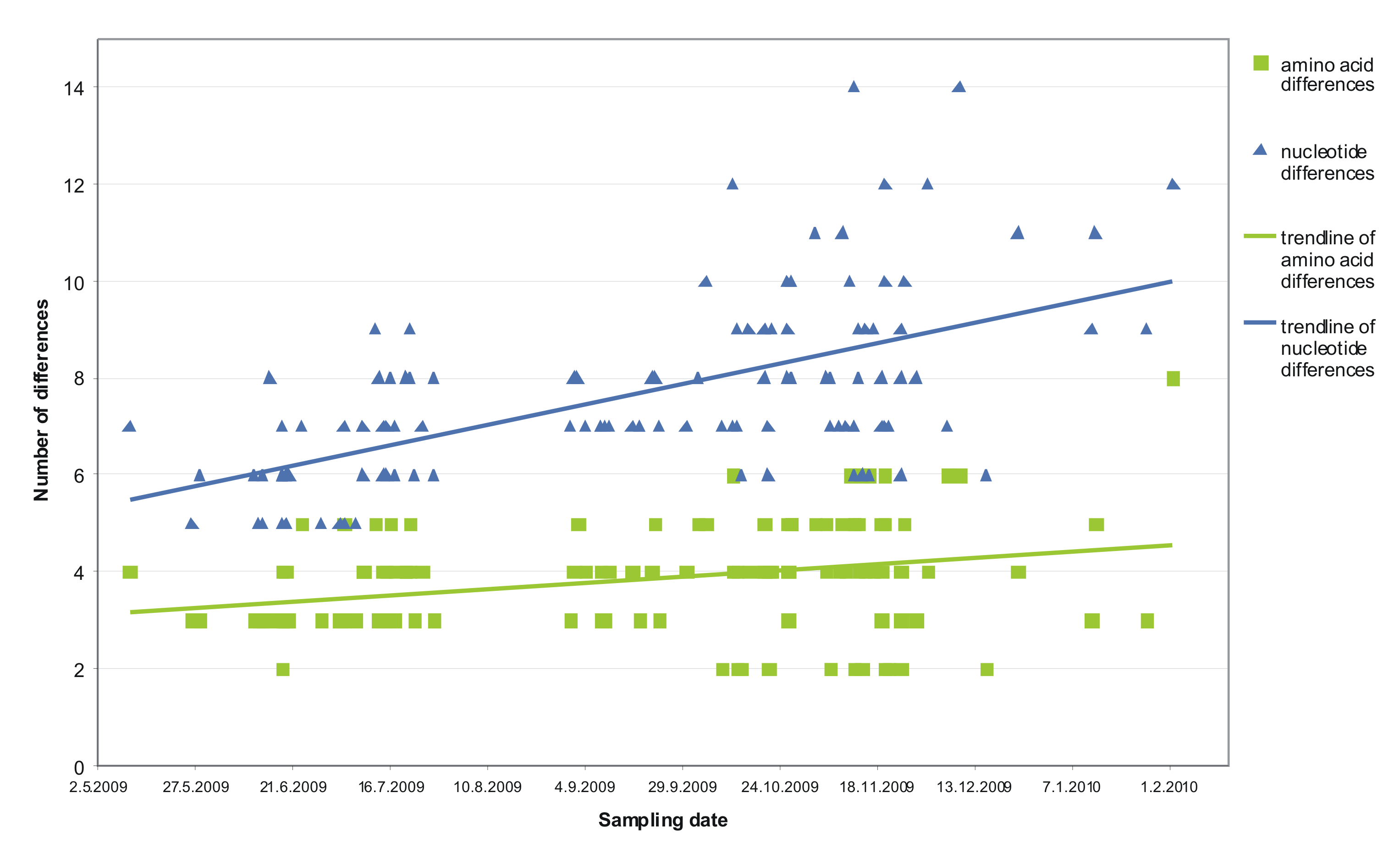

Supplement: Figure S2 — Evolutionary rate of entire HA gene. The number of nucleotide and amino acid changes between the HA of Finnish pandemic viruses and the vaccine strain A/California/07/2009 are shown as a function of time. The trend lines have been drawn using Microsoft Office Excel 2003. The number of nucleotide changes are shown in blue and in amino acid changes in green. (0.48 MB TIF) [file pone.0013329.s002.tif]
